# Identification of two Amino Acids in the C-terminal Domain of Mouse CRY2 Essential for PER2 Interaction

**DOI:** 10.1186/1471-2199-11-69

**Published:** 2010-09-14

**Authors:** Natali Ozber, Ibrahim Baris, Gulnaz Tatlici, Ibrahim Gur, Seda Kilinc, Evrim B Unal, Ibrahim H Kavakli

**Affiliations:** 1 Material Science and Engineering, College of Engineering, Koc University, Rumeli Feneri Yolu, 34450 Sariyer, Istanbul, Turkey; 2 Department of Chemical and Biological Engineering, College of Engineering, Koc University, Rumeli Feneri Yolu, 34450 Sariyer, Istanbul, Turkey; 3 Centre for Computational Biology and Bioinformatics, College of Engineering, Koc University, Rumeli Feneri Yolu, 34450 Sariyer, Istanbul, Turkey

## Abstract

**Background:**

Cryptochromes (CRYs) are a class of flavoprotein blue-light signaling receptors found in plants and animals, and they control plant development and the entrainment of circadian rhythms. They also act as integral parts of the central circadian oscillator in humans and other animals. In mammals, the CLOCK-BMAL1 heterodimer activates transcription of the *Per *and *Cry *genes as well as clock-regulated genes. The PER2 proteins interact with CRY and CKIε, and the resulting ternary complexes translocate into the nucleus, where they negatively regulate the transcription of *Per *and *Cry *core clock genes and other clock-regulated output genes. Recent studies have indicated that the extended C-termini of the mammalian CRYs, as compared to photolyase proteins, interact with PER proteins.

**Results:**

We identified a region on mCRY2 (between residues 493 and 512) responsible for direct physical interaction with mPER2 by mammalian two-hybrid and co-immunoprecipitation assays. Moreover, using oligonucleotide-based degenerate PCR, we discovered that mutation of Arg-501 and Lys-503 of mCRY2 within this C-terminal region totally abolishes interaction with PER2.

**Conclusions:**

Our results identify mCRY2 amino acid residues that interact with the mPER2 binding region and suggest the potential for rational drug design to inhibit CRYs for specific therapeutic approaches.

## Background

Circadian rhythms are oscillations in the behaviour and biochemical reactions of organisms and occur with a periodicity of approximately 24 hours [1]. Traditionally, the circadian system has been conceptualized in terms of three components: an input component, a clockwork component and an output component. Recent studies, however, have shown that there are considerable overlaps between the three components at both macroscopic and microscopic levels, such that the three components may be organized into multiple interconnecting pathways [2,3]. In mammals, the input component is mediated at the macroscopic level by the visual perception of light. The master circadian clock is located in the hypothalamus as a pair of neuron clusters that are known as the suprachiasmatic nuclei (SCN). The output component is elaborated by endocrine mechanisms in the form of prokineticin (PK2) and, transforming growth factor-α (TGF-α) secretion [4,5] from the SCN which also engage other regions of the brain through neural connections. At the molecular level, the clockwork of the SCN involves several proteins that participate in positive and negative transcriptional feedback loops. The mammalian BMAL1 and CLOCK are the transcription factors that contain two basic helix-loop-helix domains and bind E-box elements (CACGTG) in the Period and Cryptochrome clock genes and activate their transcription (the positive arm of the feedback loop) [4-6]. The PERIOD (PER) and CRYPTOCHROME (CRY) proteins act as negative regulators of transcription that is driven by the BMAL1/CLOCK heterodimer (the negative arm of the feedback loop) [4,7]. PER and CRY form ternary complexes with casein kinase Iε (CKIε) in the cytoplasm. This ternary complex translocates into the nucleus, where they acts as a negative regulator of BMAL1/CLOCK-driven transcription [6,8].

Cryptochromes were initially identified as putative blue-light photoreceptors due to their high homology at the primary amino acid level to photolyase, which repairs ultraviolet (UV)-light-induced photoproducts in DNA [9]. Comparison of the crystal structures of plant cryptochrome and photolyase has revealed that the two classes of proteins are also structurally quite similar [10]. Therefore, it is somewhat surprising that, unlike photolyase, cryptochrome does not possess DNA repair activity [11,12]. However, cryptochrome has been proposed to act as a circadian photoreceptor [11].

Alignments of various cryptochromes and photolyases have shown that cryptochromes from mammals, plants, and insects have extended C-terminal domains that range from 30 to 300 amino acids, depending on the species [13]. Studies on the Arabidopsis CRY protein have shown that the C-termini of CRYs mediate blue-light signals by interacting with COP proteins [3,14]. In addition, the Drosophila CRY protein transmits the blue-light signal by interacting with TIM proteins through its C-terminus [15]. Although the role of mammalian CRYs and their C-termini in circadian photoreception is not clear, their roles in circadian rhythms have been well established. Previous studies have indicated that mammalian cryptochromes together with PER2-CKIε inhibit BMAL1-CLOCK driven transcription [8]. Using both yeast and mammalian two-hybrid systems, it has been shown that CRY directly interacts with both BMAL1 and PER2 [16,17]. Recent studies have indicated that interaction between CRY and CLOCK and BMAL1 occur through the C-termini of CRY [18,19]. However, the CLOCK-BMAL1 interacting region of CRYs has not been defined at the amino acid level. In this study, we identified the amino acids of mCRY2 C-terminus that are important for interaction with PER2. Our results revealed that Arg-501 and Lys-503 are responsible for the interaction between mouse CRY2 and PER2 using the mammalian two-hybrid system with co- immunoprecipitation and mutagenesis.

## Results and Discussion

### Analysis of interaction between mouse CRY2 and PER2, BMAL1 and CLOCK

**Figure 1:**
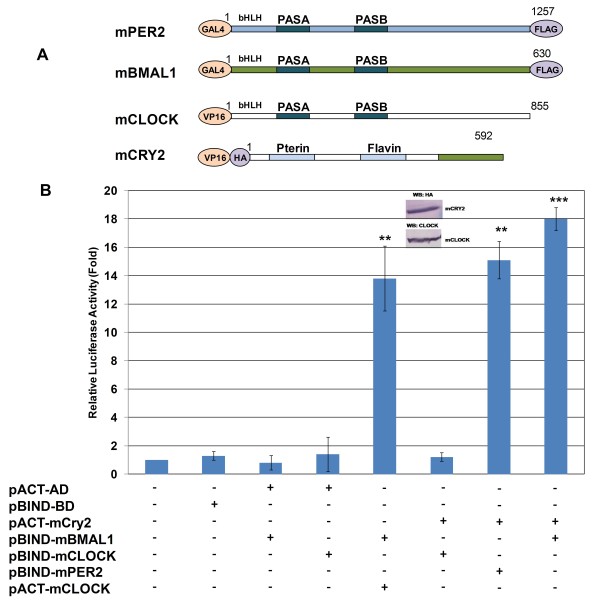
**Analysis of the interaction between mouse CRYs and various clock proteins using a mammalian two-hybrid system**. (A) Mouse Cry2 cDNA was cloned into pACT, where it is fused with the VP16 domain through its N-terminus. The cDNA of *mPer2*, *mClock*, and *mBmal1 *were cloned into pBIND and proteins were expressed with the GAL4 domain in HEK 293T cells. (B) HEK 293T cells were transfected with *pBIND-mBmal1*/*pACT-mClock*, *pBIND-mBmal1*/*pACT-mCry2 *and *pACT-mCry2*/*pBIND-mClock*, and *pACT-mCry2/pBIND-mPer2 *separately along with the pGL5*luc *reporter plasmid. Top Panel: Expression levels of HA-mCRY2 and mCLOCK analyzed by western blot. Bottom Panel: Reporter activity was examined 24 h after transfection. Relative activities were calculated based on mock samples and values were plotted. Error bars indicate SEM from at least 3 experiments. ** p < 0.005, *** p < 0.0001 as determined by student's t-test compared to the relevant controls. pACT-AD: pACT carries VP16; PBIND-BD: pBIND carries GAL4.

CRY proteins have two domains: a core region with a high similarity to photolyases and C-terminal 'tails' that vary considerably in length and sequence both between the CRYs and photolyases and among individual CRYs [20]. The N-terminal region contains conserved binding sites for two cofactors, a flavin and the pterin (Fig. 1), and functions as the photosensor in Arabidopsis and Drosophila CRYs [9]. However its precise role in mammalian CRYs remains to be defined. It is known that mammalian CRYs form a ternary complex with mPER2 and CKIε and act as negative regulators that interact with BMAL1/CLOCK.

The interaction of mammalian CRY proteins with BMAL1, CLOCK, and PER2 proteins in mouse was investigated using either a yeast two-hybrid or co-immunoprecipitation assays [16,17]. To test the feasibility of the mammalian two-hybrid system for the investigation of clock protein-protein interactions, the cDNA of *mCry2 *was cloned into pACT, where it was fused with VP16, and cDNAs of *mClock*, *mPer2*, and *mBmal1 *were cloned into pBIND, where they were fused with GAL4 (Fig. 1A). Then, *pACT-mCry2 *and *pBIND-mPer2*, *pBIND-Bmal1*, or *pBIND-mClock *were transfected into HEK 293T cells. Co-expression of VP16-mCRY2, either with GAL4-mBMAL1 or GAL4-mPER2, yielded comparable levels of luciferase activity (Fig. 1B). The co-expression VP16-mCRY2 with GAL4-mCLOCK, compared with the proper controls, resulted in no luciferase activity, which indicates that these two proteins do not physically interact within the cell (Fig. 1B). To see if this result is not due to an expression problem, we carried out western blot analysis to detect expression levels of mCRY2 and mCLOCK. As shown in Fig. 1B, both proteins are expressed at comparable level. These results are consistent with previously published results [16,19], but recent studies have shown the interaction between CRY1 and CLOCK using co-immunoprecipitation [17]. It is possible that this interaction is only able to occur upon complex formation rather than being based solely on physical interaction between these two proteins. To eliminate the possibility that the interactions of the clock proteins observed in the mammalian two-hybrid system are due to the binding and activation domains of the vectors, the cDNAs of the clock genes were also cloned into reciprocal vectors. After the transfection of various combinations of constructs into HEK 293T cells, comparable levels of luciferase activity were measured using the same amounts of DNA, in accordance with previous data (data not shown).

**Figure 2:**
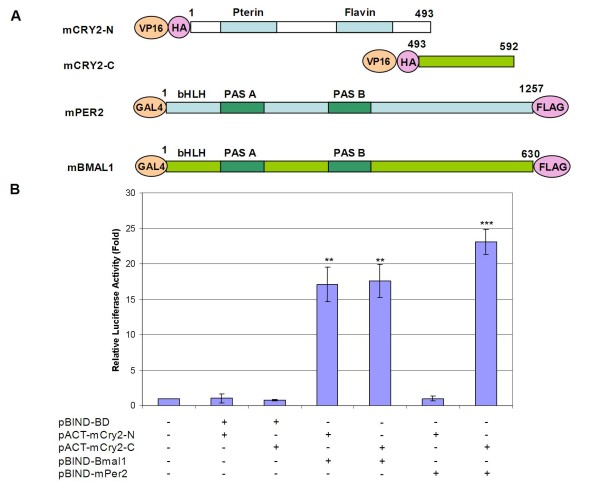
**Analysis of the interaction between mCry2 N- and C-termini with mBMAL1 and mPER2 proteins using a mammalian two-hybrid system**. **(**A) Both *mCry2 *N- and C-termini cDNA were cloned into pACT, where they are fused with the VP16 domain through their N-termini. The cDNA of *mPer2 *and *mBmal1 *were cloned into pBIND and proteins were expressed with the GAL4 domain in HEK 293T cells. (B) HEK 293T cells were transfected with the indicated constructs along with the pGL5*luc *reporter plasmid. Reporter activity was examined 24 h after transfection. Relative activities were calculated based on mock samples and values were plotted. Error bars indicate SEM from at least 3 experiments. ** p < 0.005, *** p < 0.0001 as determined by student's t-test compared to the relevant controls.

We separated *mCry2 *into their C- and N-termini (Fig. 2A) [13] by PCR and the two domains were cloned into pACT, thus fusing both CRY domains with the VP16 domain (Fig. 2A). Based on the luciferase activity, the photolyase homology region (PHR) domain of mCRY2 (first 492 amino acids) did not interact with mPER2, but did interact with mBMAL1 (Fig. 2B). These data are consistent with a previous study [19]. These findings indicate that mammalian two-hybrid is appropriate to identify the domains and perhaps amino acids that play critical roles in protein-protein interactions.

### Identification of the region on mCRY2 proteins that interact with mPER2

**Figure 3:**
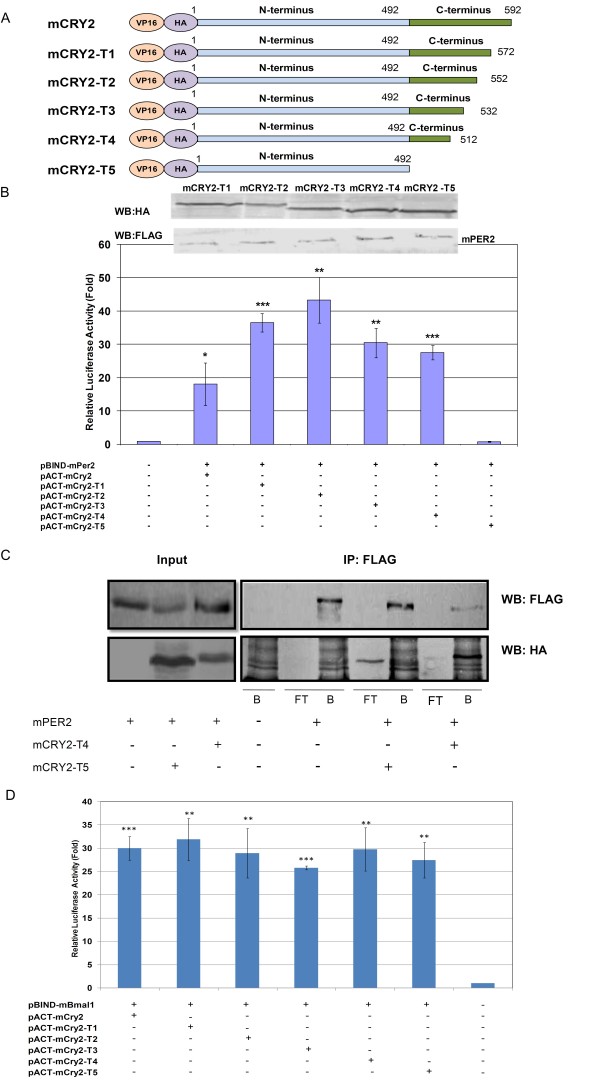
**Analysis of the interaction between mCRY2 C-terminal truncated forms with mBMAL1 using a mammalian two-hybrid system**. (A) cDNA of *mCry2 *was cloned into pACT, where it is was fused with the VP16 domain through their N-termini. (B) HEK 293T cells were transfected with *pBIND-mPer2 *and *pACT-mCry2-T1/T2/T3/T4/T5 *along with the pGL5*luc *reporter plasmid. Top Panel: Expression of HA-mCRY2 and FLAG-mPER2 analyzed by western blot. Bottom Panel: Reporter activity was examined 24 h after transfection. Relative activities were calculated based on mock samples and values were plotted. Error bars indicate SEM from at least 3 experiments. * p < 0.01, ** p < 0.005, *** p < 0.0001 as determined by student's t-test compared to the control. (C) mCRY2-T4/mPER2 and mCRY2-T5/mPER2 interaction in mammalian cells. HEK293T cells were transfected with *Flag-mPer2 *and with or without *HA-mCry2-T4 *or co-transfected with *Flag-mPer2 *and *HA-mCry2-T5*. Lysates were then immunoprecipitated (IP) with anti-Flag antibodies. Immunoprecipitates were separated by SDS- PAGE and then immunoblotted with antibodies against FLAG and HA as indicated. Left Panel: Input fractions. Right Panel: B and FT denote bound and flow through fractions, respectively. (D) HEK 293T cells were transfected with *pBIND-mBmal1 *and *pACT-mCry2-T1/T2/T3/T4/T5 *along with the pGL5*luc *reporter plasmid. Reporter activity was examined 24 h after transfection. Relative activities were calculated based on mock samples and values were plotted. Error bars indicate SEM from at least 3 experiments. * p < 0.01, ** p < 0.005, *** p < 0.0001 as determined by student's t-test compared to the control.

**Figure 4:**
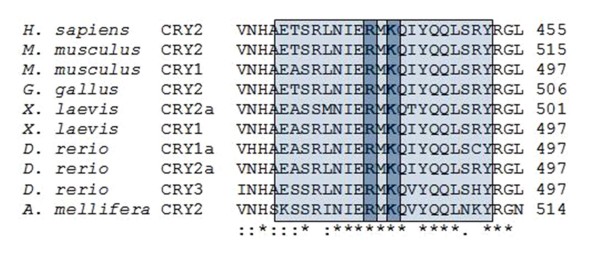
**Analysis of the conservation of Arg and Lys residues**. The protein sequence alignment of Arg-501 and Lys-503 of mCRY2 across species was performed with ClustalW2.

We wanted to identify the amino acid(s) in mCRY2 that are important for binding to mPER2. For this purpose, we cloned *mCry2 *constructs that had been generated by deletion of the C-terminus in multiples of 20 amino acids. Truncation of *mCry2 *was carried out by PCR and PCR products were cloned into the pACT vector. The resulting constructs were named *pACT-mCry2-T1 *(1-572 aa), *pACT-mCry2-T2 *(1-552 aa), *pACT-mCry2-T3 *(1-532 aa), *pACT-mCry2*-T4 (1-512 aa), and *pACT-mCry2-T5 *(1-492 aa) (Fig. 3A). Mammalian two-hybrid showed that VP16-mCRY2-T5 yielded no luciferase activity when co-expressed with GAL4-mPER2 (Fig. 3B). To ensure that this lack of activity was not an artifact of reduced expression of the proteins, the samples were subjected to western blot analysis. Western blot analysis of cell lysates from HEK 293T cells that transiently expressed FLAG- tagged GAL4-mPER2 and HA- tagged VP16-mCRY2-T1/T2/T3/T4/T5 revealed proteins of the expected molecular weight at comparable levels (Fig. 3B, upper panel). Hence, we conclude that the region between 493 and 512 of mCRY2-T4 is responsible for the interaction with mPER2. Then, we performed co-immunoprecipitation assays as a supportive method. HEK 293T cells were transiently transfected with *pACT-mCry2-T4/pBIND-mPer2, pBIND-mPer2*, and *pACT-mCry2-T5/pBIND-mPer2*. mPER2 was immunoprecipitated with FLAG antibodies as seen in Fig. 3C. FLAG-tagged mPER2 proteins pulled down HA-tagged mCRY2-T4, but FLAG-tagged mPER2 proteins did not pull down HA-tagged mCRY2-T5 protein. As seen in Fig. 3C, mCRY2-T5 was in the flow through. Other bands of molecular weights different than the expected size of CRY2 s were non-specific bands that were observed in the FLAG-mPER2 proteins or FLAG resin that passed through the HEK 293T cell-free extract. The results of mammalian two-hybrid and co-immunoprecipitation assays show that the region between 493-512 amino acids of mCRY2 proteins is required for the interaction with mPER2 protein. Additionally, we have tested the interaction between mBMAL1 and truncated mCRY2 constructs. We have shown that the absence of the region 493-512 amino acids did not prevent the BMAL1-mCRY2 interaction (Fig. 3D). This indicates that these two amino acids are important only to interact with the mPER2 and do not interfere with the interaction mCRY2-mBMAL1. In fact, the region between 493 and 512 of mCRY2 are located in the corresponding coiled-coil region of mCRY1 (Fig. 4), which was previously proposed as the interaction region of mCRY1 with mPER2 proteins [18].

### Identification of amino acid residues of mCRY2 that mediate interaction with mPER2

**Figure 5:**
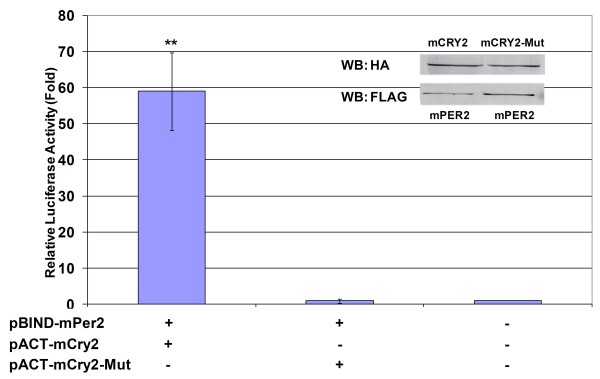
**Analysis of the interaction between full-length wildtype and mutant mCRY2 with mPER2 using a mammalian two-hybrid system**. HEK 293T cells were transfected with *pBIND-mPER2*/*pACT-mCry2 *and *pACT-mCry2-Mut*/*pBIND-mPer2 *and the pGL5*luc *reporter plasmid. Top Panel: Expression levels of HA-mCRY2 and FLAG-mPER2. Bottom Panel: Reporter activity was examined 24 h after transfection and the relative luciferase activity was calculated and plotted. Error bars indicate SEM from at least 3 experiments. ** p < 0.005 as determined by student's t-test compared to the control.

**Figure 6:**
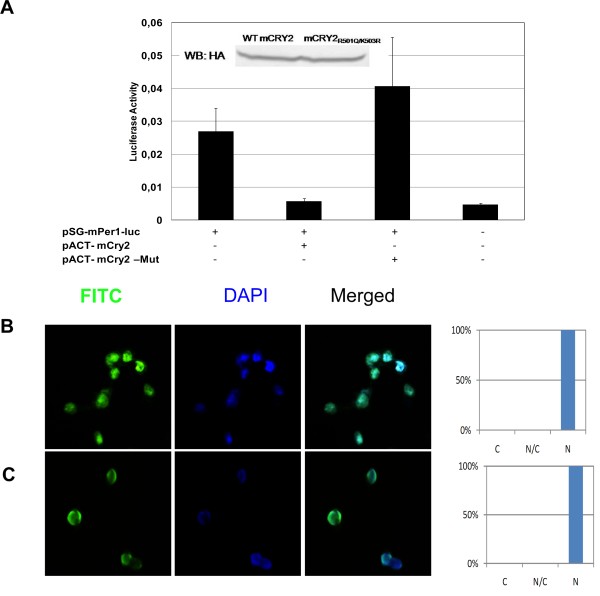
**Repression of CLOCK-BMAL1 activity by wild type but not mutant mCRY2**. (A) HEK293T cells were transfected with the *pSG-mPer1-Luciferase *construct and the indicated wildtype (WT) or mutant *mCry2 *plasmids. Averages of 3 experiments with SEM are plotted. * p < 0.05, as determined by student's t-test compared to the control (*pSG-mPer1-Luc *alone). Inset shows analysis of expression levels of WT and mutant CRY2 by western blot analysis. (B) HEK293T cells were transfected with pACT-mCry2 and (C) pACT-mCRY2_R501Q/K503R _and localizations of encoded proteins were analyzed by using immunocytochemistry with HA antisera and FITC-conjugated secondary antibodies. Both wild type and mutant cryptochromes were localized in the nucleus.

To identify the amino acid residues of mCRY2-T4 that interact with mPER2, we prepared degenerate oligos for 20 amino acids in the region between 493 and 512. We carried out PCR using *pACT-mCry2-T4 *as the template. After cloning mutants into the pACT vector, 50 colonies were randomly selected. Mutants were subjected to mammalian two-hybrid to examine the effect of mutated amino acids at various positions (Table 1). Many mutants had comparable levels of luciferase activity with respect to wildtype, while some mutants did not yield luciferase activity at all (Table 1). These mutant constructs were sequenced to identify the mutations. Interestingly, only constructs that had mutations in either Arg501 or Lys503 or both abolished luciferase activity. These results suggested that Arg501 and Lys503, located in the 20 amino acid region between 493 and 512, are important for interaction with mPER2. Thus, we performed conservation analysis on these residues using Clustal W2. We observed that the predicted coiled-coil region has a high degree of conservation and these two amino acids within this region are fully conserved even in less related species (Fig. 4). This analysis reveals the importance of these two amino acids for PER2 and CRY2 interaction. To assess whether these two amino acids of mCRY2 are solely responsible for interaction with mPER2, we replaced Arg501 and Lys503 in full-length *mCry2 *cDNA with Gln and Arg, respectively, by site-directed mutagenesis. As shown by the mammalian two-hybrid results, there was no interaction between mutant mCRY2 and mPER2 when compared with wildtype mCRY2 and mPER2 (Fig. 5). To ensure that differential expression did not prevent the detection of luciferase activity, the samples were subjected to western blot analysis using anti-HA and anti-FLAG antibodies for mCRY2 and mPER2, respectively. As shown in Fig. 5, all cDNA that were transfected into HEK 293T cells was comparably expressed, which verifies that all proteins were expressed at comparable levels. We hypothesized that if Arg501 and Lys503 residues of mCRY2 are important for interaction with mPER2 proteins, one would expect that over-expression of mutant mCRY2 would not inhibit CLOCK-BMAL1-driven transcription [21]. Expression of *pSG-mPer1-Luc *plasmid (*mPer*1 promoter with E-box) alone resulted in a 10-fold increase luciferase activity compared with the blank. This is because the endogenous clock protein binds the *Per1 *promoter of the *pSG-mPer1-luc *construct and induces expression of luciferase. Next, we transfected *pSG-mPer1-luc/pACT- mCry2 *and *pSG-mPer1-luc/pACT-mCry2-Mut *into HEK 293T cells to assess the ability of mutant mCRY2 to repress endogenous CLOCK-BMAL1-mediated transcription from the *mPer1 *promoter, which was fused with the luciferase gene in *pSG-mPer1-luc*. We observed that over-expression of wildtype mCRY2 totally suppressed CLOCK-BMAL1-driven transcription. However, mutant mCRY2 was unable to suppress CLOCK-BMAL1-driven transcription even though both wildtype and mutant mCRY2 were expressed at comparable levels (Fig. 6A). In order to see this result is not due to the aberrant nuclear translocation of mutant CRY2, we performed immunocytochemistry under the same conditions with HA antisera and FITC conjugated secondary antibodies. As shown in Figure 6B, both mutant and wild type cryptochromes were indeed localized in the nucleus. We further analyzed the repression of CLOCK/BMAL1-driven transcription using GAL4 responsive promoter using mammalian two-hybrid system [8]. Both mutant and wild type mCRY2 were cloned into pEGFP-N1 vector. Plasmid constructs that have been used in this assay are shown in Additional file 1. The over-expression of EGFP fused wild type mCRY2 repressed the CLOCK-BMAL1-mediated luciferase activity, whereas EGFP fused mutant mCRY2 exhibited significantly reduced repressive effect in NIH3T3 cells (Additional file 1). To test the effects of mutations on the subcellular localization of mCRY2, EGFP fused mutant and wildtype mCRY2 are expressed NIH3T3 cell line and subjected to the fluorescent microscopic analysis. It was shown that GFP localized in both nucleus and cytoplasm when pEGFP vector alone is expressed in NIH3T3 cells. Since GFP is a small protein it can passively diffuse into nucleus. On the other hand both mutant and wild type CRY2 fused with GFP, localized only in the nucleus (n = 200). We have observed that both wildtype and mutant mCRY2 were localized to the nucleus. This result is consistent with the result obtained using *mPer1 *promoter with E-box. Based on the Kobayashi *et al*. (1998), PKRK motif in the C-terminus is required for the nuclear localization of mCRY2 and is enough for the nuclear localization of NLS-GFP construct [22]. R501Q/K503R mutations do not seem to affect this NLS sequence and nuclear localization patterns of these proteins (Additional file 1). Therefore the inability of mutant mCRY2 to suppress transcription is not due to impaired nuclear accumulation of mCRYs as both mutant and wildtype mCRY2 localized to the nucleus. Thus, we conclude that Arg501 and Lys503 of mCRY2 are not only necessary for interacting with mPER2 but are also essential for suppressing CLOCK/BMAL1 activity. This finding suggests the potential for rational drug design to inhibit cryptochrome for specific therapeutic approaches [23].

**Table 1:** Random mutagenesis of residues between 493-512 in the mCry2-T4 construct and relative luciferase results.

																					Luciferase Assay
**mCry2-T4**	**E**	**T**	**S**	**R**	**L**	**N**	**I**	**E**	**R**	**M**	**K**	**Q**	**I**	**Y**	**Q**	**Q**	**L**	**S**	**R**	**Y**	**+++++**

M1					P		T		**Q**				T		X		P				-
M2	G				P				**Q**		**R**		T				P		R		-
M3					P		T						T		X		P				++
M4			G		P		T						T						R		+++
M5			G		P		T		**Q**		**R**		T				P		R		-
M6	G				P		T		**Q**				T				P				-
M7							T		**Q**												-
M8							T				**R**		T				P				-
M9	G		G		P		T						T						R		++
M10					P		T						T				P				+++
M11	G				P						**R**		T				P				-
M12					P		T						T						R		++
M13			G		P		T		**Q**		**R**		T				P		R		-
M14			G		P		T				**R**		T				P				-
M15							T		**Q**				T				P		R		-
M16					P		T		**Q**				T				P				-
M17										T											++++
M18				Q		D				T								P			++++
M19								G		T								P			++++
M20				Q										H						H	++++
M21		I		Q		D				T				H		X		P		H	+++
M22				Q		D				T				H				P			++++
M23				Q								X		H				P		H	++
M24		I								T				H		X					+
M25								G						H						H	+
M26				Q		D				T				H		X				H	++

Only mutated positions are listed. × indicates the stop codon.

## Conclusions

Our work shows that Arg-501 and Lys-503 of mCRY2 are required not only for interaction with mPER2 but are also important for the suppression of BMAL1-CLOCK-mediated transcription. Our results identify the amino acid residues of mCRY2 that interact with the mPER2 binding region and may help to identify the new genetically inherited circadian abnormalities in humans.

## Methods

### Bacterial Strains and Plasmids

The *E. coli *DH5α strain was used as the host strain for subcloning and manipulation of the cDNA of *mCry2*, *mPer 2*, *mBmal1 *and *mClock *genes. The mammalian two-hybrid vectors, pBIND and pACT, were purchased from Promega (Wisconsin, USA). pcDNA4/myc-His A was purchased from Invitrogen (California, USA). The bacterial strains were maintained at -80°C in Luria-Bertani (LB) broth supplemented with 15% (v/v) glycerol. Cultures were grown at 37°C in LB containing ampicillin at 100 μg/ml where indicated.

### Plasmid Construction

All the cDNAs were amplified by PCR (with Pfu Turbo DNA Polymerase) using appropriate primers that contained NotI and EcoRV restriction enzyme recognition sites. After digestion of the PCR products, the genes were subcloned into pBIND, pACT, and pcDNA4/myc-His A mammalian expression vectors. The gene sequences of newly made constructs were determined to ensure the constructs did not contain mutations that had been introduced during the PCR amplification.

### Mutagenesis

Random mutagenesis of the last 20 amino acids region (493-512 aa) of the *pACT-mCry2-T4 *construct was performed with degenerate reverse primers. The following primers were used to mutate the odd or even numbered residues, respectively: 5'- ggtacctgcggccgcGTRTCTCGRC AGCTRTTGGTRGATCTRCTTCRTTCGTYCAATGTYGAGCYGACTARTCTCtgcatgattgacgat-3' or 5'- ggtacctgcggccgcGTAYCTCGA-CRGCTGTTRGTA GRTCTGCYTCATTYG TTCARTGTTGRGCCGACYAGTCYCtgcatgattgacgat 3'. Site-directed mutagenesis for residues 501 and 503 was introduced by PCR. Plasmids *pACT-mCry2 *or *pACT-mCry2-T4 *were used as templates. Using the primers Forward: 5' CAACATTGAACAAATGAGGCA GATCTACCAAC 3' and Reverse: 5' GTTGGTAGATCTGCCTCATTTGTTCAATGTTG 3', PCR was performed in a total volume of 50 μl containing approximately 50 ng of plasmid samples, 20 pmol of each primer, 0.2 mM dNTPs, and 2.5 unit Dream Taq DNA polymerase (MBI Fermentas). Conditions for the 18 cycles of amplification were 95°C for 30 s, 50°C for 30 s and 68°C for 14 min. Before the first cycle, reaction mixtures were kept at 95°C for 4 min and at the end of the 18th cycle an additional 68°C extension period was applied for 10 min. Samples were then treated with DpnI restriction enzyme to remove the template DNA and transformed into *E coli*. Transformed cells were plated and selected on appropriate selective media. Mutagenesis results were verified by DNA sequencing carried out by the Burc Laboratory (Istanbul, Turkey).

### Cells, Transfections and Luciferase Assay

HEK 293T cells were grown in Dulbecco's modified Eagle's medium (Invitrogen) supplemented with 10% fetal bovine serum. The day before transfection, 4 × 10^5 ^cells were seeded and then total 2 μg of equimolar amount of plasmid DNA constructs were transfected into the cells with HD Fugene (Roche, California, USA) in six-well plates according to the manufacturer's instructions. The preparation of cell lysates and dual luciferase assay were carried out according to the manufacturer's protocol (Promega). After 24 h, activity of both firefly and *Renilla *luciferase was measured using a luminometer. The firefly luciferase activity was normalized using *Renilla *luciferase for transfection efficiency. All experimental results are the average of at least three experiments.

### Western Blot Analysis

Anti-HA and anti-FLAG antibodies were purchased from Santa Cruz (CA, USA) and Sigma (MO, USA), respectively. Total protein (100 μg), extracted from the cells as described previously, was separated by 8% SDS-PAGE and transferred onto a polyvinyldifluoride membrane. The membrane was blocked with 5% BSA and incubated with mouse anti-HA antibody and anti-FLAG antibody for 1 h at room temperature. The blots were incubated with the appropriate secondary antibody - alkaline phosphatase-conjugated anti-mouse - and the blots were developed with NBT and BCIP Western blotting detection system.

### Nuclear Localization Studies

HEK293T cells were grown on coverslips and cultured in DMEM supplemented with 10%FBS. Cells were seeded at 2 × 10^5 ^density 20 h prior to transfection and transfected with pACT-Cry2-HA and pACT-Cry2-HA by Fugene HD (Roche, IN). Transfected cells were fixed with 4% paraformaldehyde for 10 minutes, permeabilized with 0.3% Triton-X. Permeabilized coverslips were blocked in 10% normal goat serum for 1 hour and incubated with monoclonal mouse anti-HA antibody (1:500) (Santa Cruz) for 1 hour. After incubation with goat anti-mouse (H+L) FITC conjugated antibody (1:200) (Invitrogen) for 1 hour, nuclei were stained with DAPI. Coverslips are mounted onto glass slides and fluorescent images are generated by Zeiss Axioplan 2 fluorescent microscope. 200 blind counts of transfected cells were performed to generate the statistical data.

NIH3T3 cells were grown on coverslips and cultured in DMEM+10%FBS. Cells were seeded at 1 × 10^5 ^density at 6-well plates 24 h prior to transfection. Both wild type and mutant Cry2 genes were cloned into pEGFP-N1 (Clontech, CA) and constructs were transfected by Fugene HD (Roche, IN) transfection reagent according to the manufacturer's instructions. The transfected cells were fixed with 4% paraformaldehyde for 10 minutes, washed with PBS and subsequently stained with DAPI. Fluorescent images were generated by Leica confocal microscope. Localizations of the proteins were assessed by microscope and 200 blind counts of transfected cells were performed to generate the statistical data.

## Authors' contributions

NO carried out the cloning of truncated forms of *mCry2*, performed random mutagenesis, the sequence alignment and the statistical analysis, participated in luciferase assays, western blot analysis and drafted the manuscript. IB performed site directed mutagenesis, helped to characterize the mutants and draft the manuscript. GT performed transfections, luciferase assays and western blot analysis. IG carried out the nuclear localization study. SK performed the co-immunoprecipitation. EBU carried out the cloning of *mCry2 *to pEGFP-N1. IHK designed and coordinated the study and wrote the manuscript. All authors read and approved the final manuscript.

## Supplementary Material

Additional file 1 **Figure S1: Analysis of subcellular localization of the wild type and mutant Cry2 proteins in NIH3T3**. (A) Top Panel: Both wild type and mutant *mCry2 *cDNA were cloned into pEGFP-N1 A where they were fused to EGFP through their N-termini. The cDNA of *mClock *was cloned into pACT, where it is fused with the VP16 domain through its N-termini and the cDNA of *mBmal1 *was cloned into pBIND, where it is fused with the GAL4 domain through its N-termini. Bottom Panel: NIH3T3 cells were transfected with *pBIND-mBmal1*/*pACT-mClock*, *pBIND-mBmal1*/*pACT- mClock/pEGFP-mCry2 *and *pBIND-mBmal1*/*pACT- mClock/*p*EGFP-mCry2-Mut *separately along with the pGL5*luc *reporter plasmid. Reporter activity was examined 24 h after transfection and the relative luciferase activity was calculated and plotted. Error bars indicate SEM from at least 3 experiments. * p < 0.05, as determined by student's t-test compared to the wild type inhibition. (B)In order to assess the subcellular localization of the Cry2 proteins, NIH3T3 cells were transfected with pEGFP, pEGFP-mCry2 and pEGFP-mCry2_R501Q/K503R _constructs. The EGFP fluorescence was observed as a diffuse nuclear/cytoplasmic localization, whereas both wild type and mutant Cry2 proteins were localized in nucleus. There was not any significant perturbation in the nuclear localization of mutant Cry2 with respect to wild type protein as quantified with 200 blind counts of the transfected cells. Click here for file
