# Supplementary material for: Identification of two Amino Acids in the C-terminal Domain of Mouse CRY2 Essential for PER2 Interaction
Source: BMC Mol Biol. 2010 Sep 14;11:69. doi: 10.1186/1471-2199-11-69 (PMC2944120; doi:10.1186/1471-2199-11-69)

**Figure S1-Analysis of subcellular localization of the wild type and mutant Cry2 proteinsin NIH3T3.** (A) Top Panel: Both wild type and mutant *mCry2* cDNA were cloned into pEGFP-N1 A where they were fused to EGFP through their N-termini. The cDNA of *mClock* was cloned into pACT, where it is fused with the VP16 domain through its N-termini and the cDNA of *mBmal1* was cloned into pBIND, where it is fused with the GAL4 domain through its N-termini. Bottom Panel: NIH3T3 cells were transfected with *pBIND-mBmal1*/*pACT-mClock*, *pBIND-mBmal1*/ *pACT- mClock/* *pEGFP-mCry2* and *pBIND-mBmal1*/ *pACT- mClock/* p*EGFP-mCry2-Mut* separately along with the pGL5*luc* reporter plasmid. Reporter activity was examined 24 h after transfection and the relative luciferase activity was calculated and plotted. Error bars indicate SEM from at least 3 experiments. * p < 0.05, as determined by student's t-test compared to the wild type inhibition. (B)In order to assess the subcellular localization of the Cry2 proteins, NIH3T3 cells were transfected with pEGFP**,** pEGFP-mCry2 and pEGFP-mCry2_R501Q/K503R_ constructs. The EGFP fluorescence was observed as a diffuse nuclear/cytoplasmic localization, whereas both wild type and mutant Cry2 proteins were localized in nucleus. There was not any significant perturbation in the nuclear localization of mutant Cry2 with respect to wild type protein as quantified with 200 blind counts of the transfected cells.


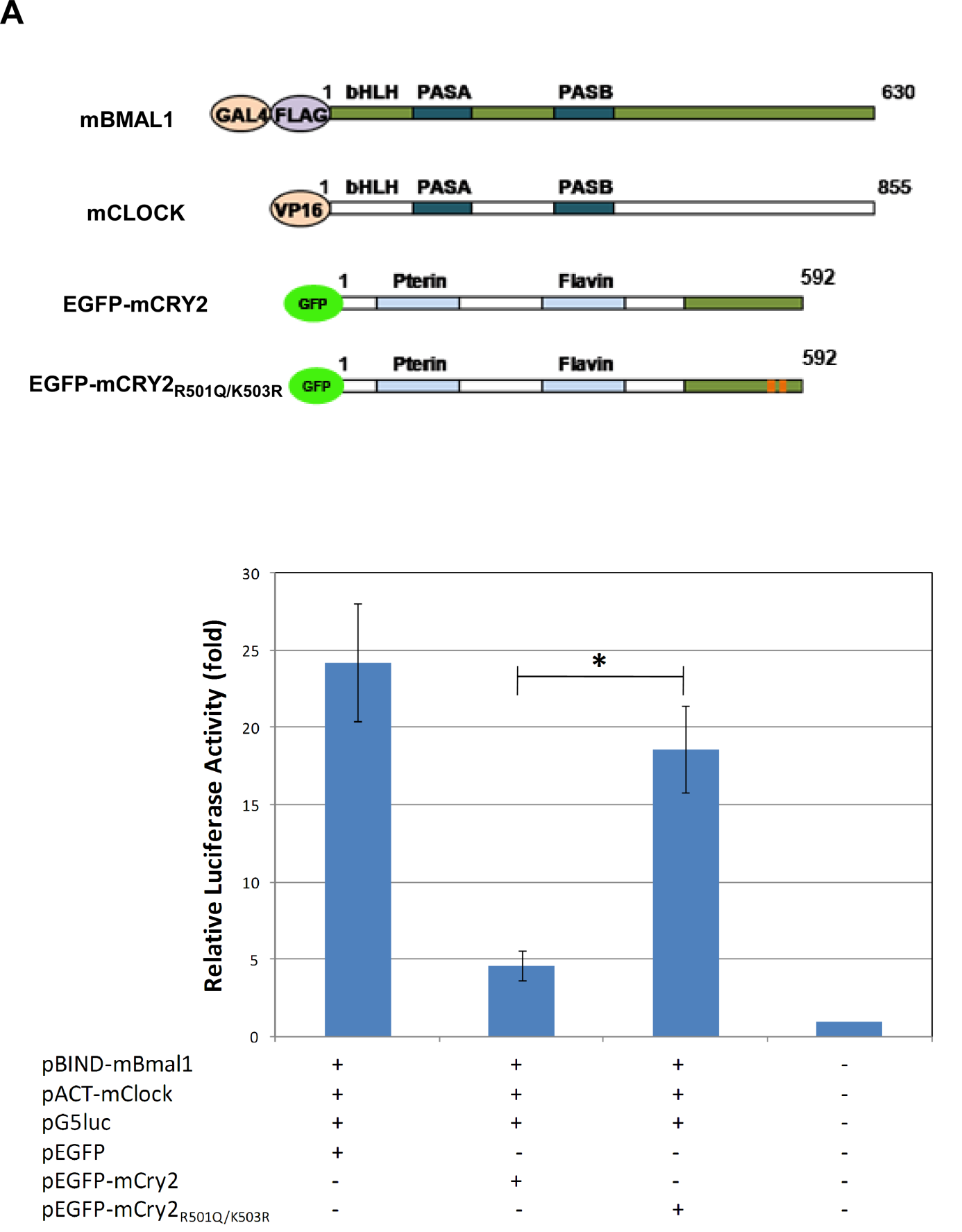


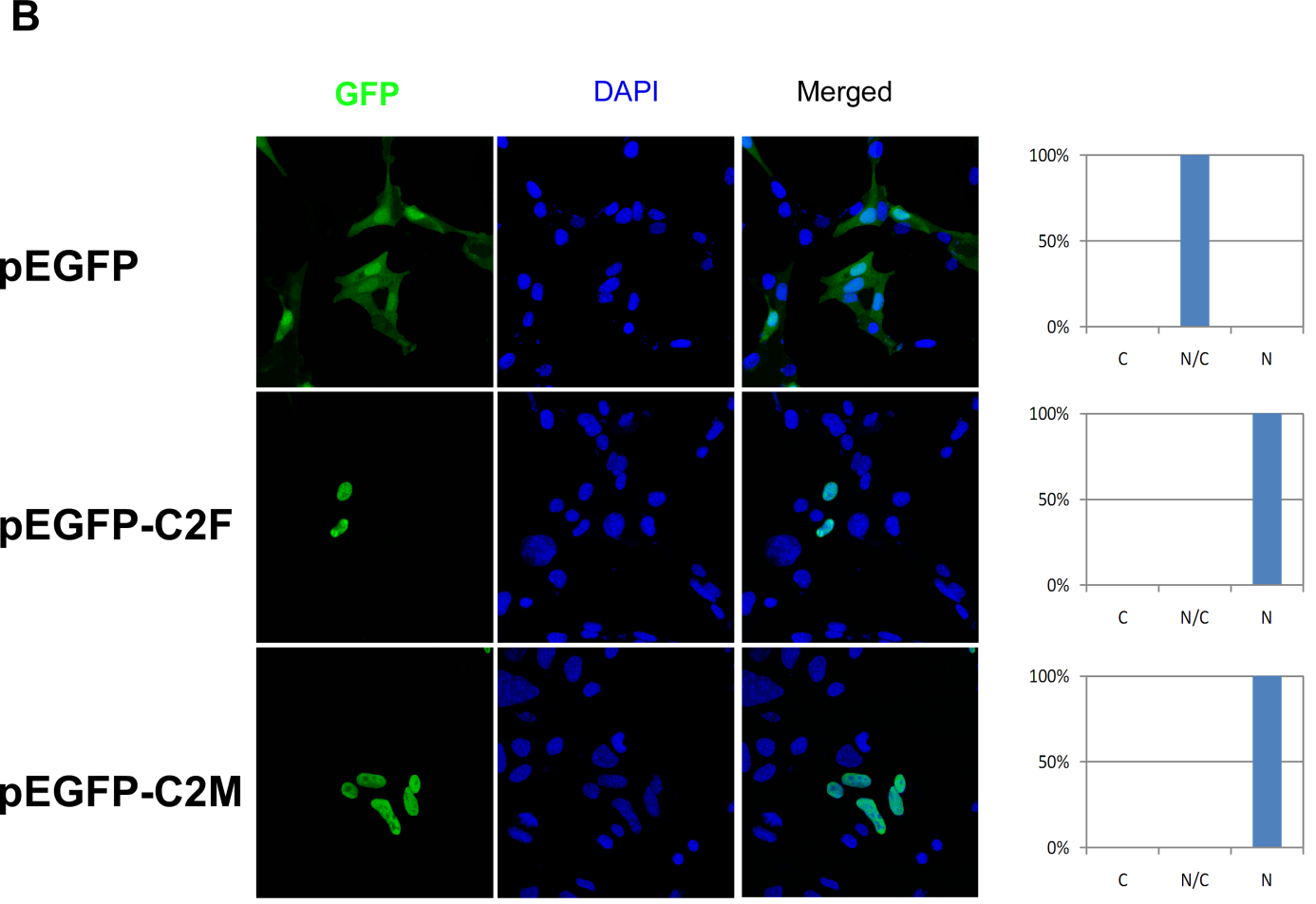

Supplement: Additional file 1 — Figure S1: Analysis of subcellular localization of the wild type and mutant Cry2 proteins in NIH3T3. (A) Top Panel: Both wild type and mutant mCry2 cDNA were cloned into pEGFP-N1 A where they were fused to EGFP through their N-termini. The cDNA of mClock was cloned into pACT, where it is fused with the VP16 domain through its N-termini and the cDNA of mBmal1 was cloned into pBIND, where it is fused with the GAL4 domain through its N-termini. Bottom Panel: NIH3T3 cells were transfected with pBIND-mBmal1/pACT-mClock, pBIND-mBmal1/pACT- mClock/pEGFP-mCry2 and pBIND-mBmal1/pACT- mClock/pEGFP-mCry2-Mut separately along with the pGL5luc reporter plasmid. Reporter activity was examined 24 h after transfection and the relative luciferase activity was calculated and plotted. Error bars indicate SEM from at least 3 experiments. * p < 0.05, as determined by student's t-test compared to the wild type inhibition. (B)In order to assess the subcellular localization of the Cry2 proteins, NIH3T3 cells were transfected with pEGFP, pEGFP-mCry2 and pEGFP-mCry2R501Q/K503R constructs. The EGFP fluorescence was observed as a diffuse nuclear/cytoplasmic localization, whereas both wild type and mutant Cry2 proteins were localized in nucleus. There was not any significant perturbation in the nuclear localization of mutant Cry2 with respect to wild type protein as quantified with 200 blind counts of the transfected cells. [file 1471-2199-11-69-S1.DOC]
